# Validation of an abbreviated version of the Lubben Social Network Scale (“LSNS-6”) and its associations with suicidality among older adults in China

**DOI:** 10.1371/journal.pone.0201612

**Published:** 2018-08-02

**Authors:** Qingsong Chang, Feng Sha, Chee Hon Chan, Paul S. F. Yip

**Affiliations:** 1 Department of Social Work and Social Administration, Faculty of Social Sciences, The University of Hong Kong, Hong Kong SAR, China; 2 Hong Kong Jockey Club Center for Suicide Research and Prevention, The University of Hong Kong, Hong Kong SAR, China; University of Queensland, AUSTRALIA

## Abstract

**Objective:**

This present study aims to estimate the structural validity, internal consistency reliability of the LSNS-6 and examine the associations between the LSNS-6 and suicidal outcomes among mainland Chinese older adults.

**Methods:**

This validation study used a big representative sample (N = 2819) of older adults in Beijing from the Sample Survey on Aged Population in Urban/Rural China. Confirmatory factor analyses (CFA) were applied to examine the factor structures of the Chinese version of LSNS-6. Internal consistency reliability of the LSNS-6 was examined by Cronbach’s alpha coefficient and the corrected item-total correlation. Logistic regression analyses were used to explore the associations between the LSNS-6 and late-life death wishes, suicidal ideation in mainland Chinese.

**Findings:**

This present study showed good internal consistency and consistent factor structure of the LSNS-6 as well as its subscales. The present data demonstrated the LSNS-6 could be a useful tool for assessing social networks among older mainland Chinese. Interestingly, among the mainland Chinese, late-life suicidality was highly associated with the LSNS-6 family subscale, rather than the friends subscale.

**Conclusion:**

The LSNS-6 could be a useful tool for assessing social networks among older mainland Chinese. In addition, suggestion is made to improve social networks, especially in family bonds and support, as a promising strategy in reducing late-life suicide risks in mainland China.

## Introduction

Social network is a multidimensional facet, referring to a web of interpersonal relationships and characteristics [1, 2]. To be more specific, social networks are the structural aspects of various social relationships characterized by size, density, boundedness, and homogeneity, and they influence individuals’ psychosocial mechanisms such as social support, influence, engagement, or access to resources [2]. Individuals feel socially connected through interactions with others in the context of social networks and are influenced by norms and values of the networks [3]. A large body of evidence suggests that social networks are associated with human health. People with fewer social network ties have been found to have an elevated risk of mortality and morbidity, suicidality, several diseases, prolonged postsurgical recovery, disability, etc. [1, 4–8].

Owing to the important role of social networks in late-life health, it is imperative to develop reliable and valid instruments to screen social isolation. The Lubben Social Network Scale (LSNS) is one of the widely-used instruments to assess perceived social support received from family and friends [9], and has been commonly applied in social and health care researches [10]. The original LSNS included 10 items and was later revised to a 12-item scale named as the LSNS-R. Due to the researchers’ use of various abbreviated versions of the LSNS-R, Lubben and Gironda created a standardized abbreviated version of the LSNS-R, the LSNS-6, employing the 6 items with the strongest loadings from the LSNS-R. Several cross-national and cross-cultural validation studies have demonstrated that the LSNS-6 was a good tool to screen for social isolation among community-dwelling older adults. The LSNS-6 demonstrated high levels of internal consistency, stable factor structures, and high correlations with criterion variables. In practice, the LSNS-6 would be more appropriate than longer instruments as a screener for social isolation [10–12].

However, use of the LSNS-6 in non-English speaking groups are scant, especially in the Chinese community. Based on previous researches, ethnicity is related to patterns of social networks [13]. Lack of culturally and linguistically appropriate measures may lead to limitations in understanding the role of social networks in the population and further delay development of culturally appropriate interventions for the non-English speaking minority populations [11]. China has become a rapidly aging country, and the proportion of older adults (aged 60 or above) would increase from 14% in 2016 to close to a third of China’s population by 2050, accounting for a quarter of the world’s aging population [14]. Without any validation, bias would be caused in measuring social networks by the LSNS-6 directly among the mainland elderly Chinese.

So far, the LSNS-6 has never been validated in the Chinese communities, and only the LSNS-R has been translated into Chinese and validated in the Hong Kong elderly [15]. However, characteristics of the elderly social network in mainland China are obviously different from those in Hong Kong. According to a comparative study of Beijing and Hong Kong, though the two Chinese cities share a common heritage of Confucian cultural traditions, they differ in degrees of economic modernization and urbanization, and in social organization of work and community life, which lead to differences between the two cities in social support networks and mental health in later life [16]. The distinct cultural values and experiences of each ethnic group create unique networks of social relationships between the mainland and Hong Kong Chinese. In addition, the previous Chinese validation study in Hong Kong was conducted by using a less representative and small sample of 91 subjects, which could limit the generalizability of the findings to the aging population at large in mainland China.

Moreover, the LSNS has been found to be associated with a wide range of health indicators such as mortality, physical health problems, depression and other mental health problems, and lack of adherence to good health practices [10, 12]. However, associations between social networks measured by the LSNS-6 and elderly suicidality were seldom studied among existing studies. Thus far, associations between the LSNS-6 and late-life suicidal outcomes such as death wishes, suicidal ideation are still unknown in Chinese communities.

Therefore, to fill in the research gaps among the existing researches, this study firstly tested the psychometric validity of the abbreviated version of the Lubben Social Network Scale (LSNS-6) among the elderly mainland Chinese by a large representative sample. Secondly, this study aims to examine the associations between the LSNS-6 and late-life suicidal outcomes after validation of the LSNS-6 among the elderly mainland Chinese.

## Materials and methods

### Sample and data source

This validation study used the Beijing subgroup data from the Sample Survey on Aged Population in Urban/Rural China (SSAPUR) conducted by the China Research Center on Aging (CRCA) in 2010. The survey was based on a stratified, multistage, and random sample design. Within the Beijing subgroup data, there were 96 urban residential communities and 5 rural villages selected randomly from the list with equal probability in Beijing. In the case of households with more than one person aged 60 years or older, one individual was selected at random. Potential participants were contacted and consent requested from them. Unavailable subjects (those who declined to participate, had illness or dementia, or were absent from home or had relocated) were replaced by older adults in the households next to those originally chosen based on the Kish table [17]. To ensure the quality of the interviews, CRCA researchers and personnel provided intensive training to field interviewers and supervised the interview process. Upon completion, questionnaires were examined on-site by the interviewers and off-site by their supervisors to ensure completion of the questionnaires to minimize missing data. All valid questionnaires were returned to the CRCA for further review and data entry. The response rate of the SSAPUR was 99.9% [17]. For this validation study, we included the samples with valid responses to the LSNS-6. Finally, the included sample size for this study was 2819 individuals aged 60 years or older.

### Measurement of variables

#### The LSNS-6

The LSNS-6 includes 6 items which measures the size of active and intimate networks of family and friends with whom they could talk to or call for help. The LSNS-6 is constructed from a set of three questions which assess kinship ties, and a comparable set of three questions which assess friend ties. The items related to kinship include 3 questions: (1) How many relatives do you see or hear from at least once a month? (2) How many relatives do you feel close to such an extent that you could call on them for help? (3) How many relatives do you feel at ease with that you can talk to about private matters? Those three questions are repeated with respect to non-kin ties by replacing the word relatives with the word friends. Every question has 5 same options as answers: 0 = none, 1 = one, 2 = two, 3 = three or four, 4 = five through eight, and 5 = nine or more [10]. The maximum score of the LSNS-6 is 30, which is an equally weighted sum of the six items. A LSNS-6 Family subscale is constructed from the three LSNS-6 questions on relatives. Similarly, a LSNS-6 Friends subscale is constructed from the three questions on friends.

#### Suicidal outcomes

In our study, suicidality was measured by death wishes and suicidal ideation in the last 1 year. In SSAPUR, the question for 1-year death wish was “Did you have thoughts of death or wishes to die in the last year?” This variable was binary, Yes = 1 and No = 0. Among the 2819 people who answered the question, 85 reported death wishes, accounting for 3.1%. Suicidal ideation was asked as “Have you ever seriously considered killing yourself in last 1 year?” This variable is also the dichotomous variable, Yes = 1 and No = 0. Among the included samples who answered the first question, 34 reported suicidal ideation, accounting for 1.2%.

#### Confounding variables

Gender was a binary variable (1 = male, 0 = female). Age was a continuous variable. Education status was measured with three categories (primary & below = 1; secondary = 2; associate & above = 3). Residence status was measure dichotomously (1 = rural; 0 = urban). Self-rated financial strain was measured using a five-point Likert scale from 1(good enough) to 5(very difficult). Self-rated health status was measured using a five-point Likert scale from 1 (very bad) to 5 (very good).

### Data analyses

Descriptive analysis was conducted using the SPSS version 18.0 including simple counting, percentages, mean values, standard deviations, and missing values to describe the demographic and other characteristics of the sample. In the following analyses, missing data were managed by the means of Listwise Deletion.

Confirmatory factor analyses (CFA) were applied to examine the factor structures of the Chinese version of LSNS-6 by AMOS 18.0. CFA is a structural equation modeling technique used to test the measurement of latent and observed variables [18] and is commonly used to validate psychometric properties of measure [19]. We examined the goodness of fit between the hypothesized model and the sample data in our study. Several indices such as Chi Square (χ^2^), the Comparative Fit Index (CFI), Root Mean Square Error of Approximation (RMSEA), and the Tucker-Lewis index (TLI) were used to assess the adequacy of model fit. According to the previous criteria, CFI greater than 0.9, RMSEA close to or smaller than 0.08, and TLI greater than 0.90 represent the good model fit [11]. Before conducting the CFA, the assumption of normality was tested by the skewness and kurtosis for each item.

Internal consistency reliability of the LSNS-6 was examined by Cronbach’s alpha coefficient and the corrected item-total correlation. It is acceptable if Cronbach’s alpha might be greater than 0.70 [20]. The corrected item-total correlation is the correlation between each item and the sum of the other items in a scale [21]. Each item in a scale should contribute to measuring a core common construct of the scale, otherwise it may be excluded. Corrected inter-item correlations using the criterion of 0.3 or higher were used to identify items related to the full scale [22, 23].

Logistic regression analyses were used to explore the associations between the LSNS-6 and late-life death wishes, suicidal ideation in mainland Chinese. Odds Ratios (ORs) smaller than 1.00 were used to report the decreased likelihood of suicidal risk in the elderly participants with better social networks. Several confounders such as age, gender, educational levels, marital status, self-rated financial status, and self-rated health status were controlled in the adjusted regression model.

## Results

### Sample characteristics

Among the 2819 elderly samples, 48.7% were males and the mean age was 70.8 years (SD = 7.69), which ranged from 60 to 96 years old. Table 1 showed the descriptive analyses on the characteristics of the samples.

**Table 1:** Characteristics of samples.

Variables	N (%)	Missing data (%)	Mean ± SD	Range
Age	2819	0	70.80 ± 7.69	60–96 (years)
Male	1372 (48.7)	0		
Education	2814	0.2		
*primary & below*	1189 (42.3)			
*secondary*	1210 (43.0)			
*associate & above*	415 (14.7)			
Marital Status	2814	0.2		
*married/cohabited*	2122 (75.4)			
*widowed*	643 (22.9)			
*divorced/others*	49 (1.7)			
Self-rated financial status	2809	0.4	3.00±0.73	1(good enough) to 5(very difficult)
Self-rated health status	2815	0.1	3.21±0.84	1(very bad) to 5(very good)
LSNS-6	2819	0	12.52 ± 7.03	0–30
LSNS-6 Family Subscale	2819	0	7.72 ± 3.93	0–15
LSNS-6 Friends Subscale	2819	0	4.80 ± 4.92	0–15
Death Wishes	85 (3.1)	2.5		
Suicidal Ideation	34 (1.2)	2.6		

LSNS-6, the abbreviated version of the Lubben Social Network Scale.

### Structural validity

**Fig 1:**
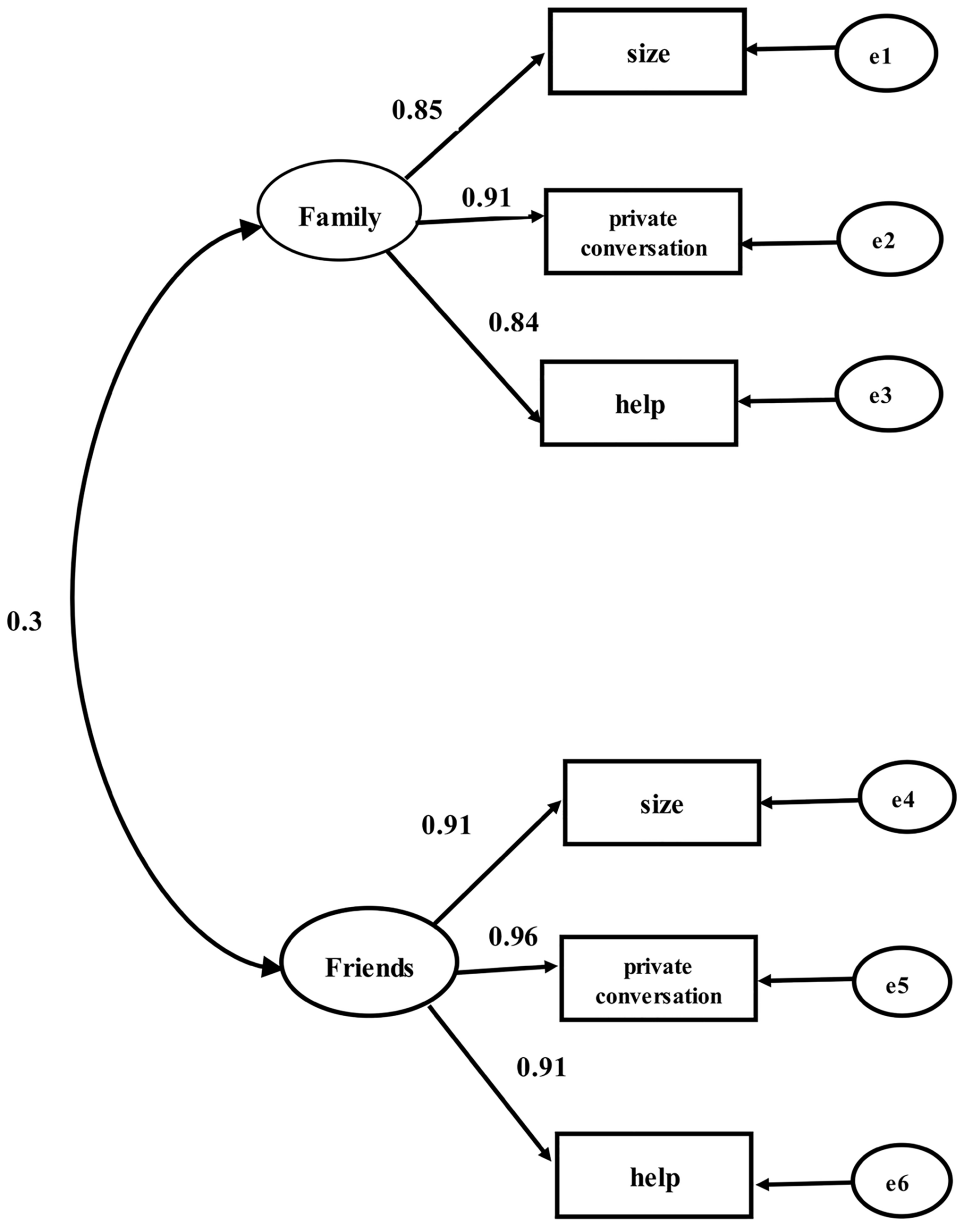
Factor structure of the LSNS-6. Note: e represents the measurement error for each item.

Table 2 illustrated the goodness of model fit indices and factor loadings of the LSNS-6. The CFA of the Chinese version of the LSNS-6 produced a very good model fit (χ^2^ = 175.33, df = 8, CFI = 0.99, TLI = 0.98, RMSEA = 0.08). Fig 1 showed the three items 1–3 related to family all loaded heavily on the family factor, and factor loadings ranged from 0.84 to 0.91. Similarly, the other 3 items 4–6 also had heavy factor loadings on the friend factor, which ranged from 0.91 to 0.96. The item with the strongest loading among all the 6 items was item 5, on the friend factor (loading = 0.96). CFA showed the two factors, family and friends, together explained about 86.8% of the variance. All the items were significant at p<0.001. Before conducting the CFA, the assumption of normality was tested by the skewness and kurtosis for each item. The greatest absolute values of skewness and kurtosis for an item were 0.63 and 1.32, respectively, indicating that the normality assumption was adequately met based on the Kline’s rule of thumb (i.e., skew index absolute value <3; kurtosis index absolute values <10) [18].

**Table 2:** Goodness of model fit indices and factor loadings of the LSNS-6.

LSNS-6 Items	Factor Loading
Item 1: How many relatives do you see or hear from at least once a month?	0.85
Item 2: How many relatives do you feel at ease with that you can talk to about private matters?	0.91
Item 3: How many relatives do you feel close to such an extent that you could call on them for help?	0.84
Item 4: How many friends do you see or hear from at least once a month?	0.91
Item 5: How many friends do you feel at ease with that you can talk to about private matters?	0.96
Item 6: How many friends do you feel close to such an extent that you could call on them for help?	0.91
**Goodness of model fit**	
χ^2^ (*df)*	175.33(8)
CFI	0.99
TLI	0.98
RMSEA	0.08

LSNS-6, the abbreviated version of the Lubben Social Network Scale. CFI = comparative fit index; TLI = Tucker-Lewis index; RMSEA = root mean square error of approximation.

### Internal consistency reliability

Cronbach’s alpha coefficient and the corrected item-total correlation were used to examine the internal consistency reliability of the LSNS-6. Table 3 illustrates the internal consistency and corrected item-total correlation of the Chinese version of the LSNS-6 validated among the elderly mainland Chinese. It would be reliable if Cronbach’s alpha were to be higher than 0.70 [20]. The Chinese version of the LSNS-6 demonstrated sound internal consistency (Cronbach’s α = 0.83). In addition, those two subscales of the LSNS-6 also had reliable internal consistency. Cronbach’s α were 0.90 and 0.95 for family subscale and friends subscale, respectively.

**Table 3:** Internal consistency and corrected item-total correlation.

	LSNS-6	Family Subscale	Friends Subscale
**Family Subscale**			
Item 1: Size	0.50	0.79	
Item 2: Private Conversation	0.56	0.83	
Item 3: Help	0.54	0.78	
**Friends Subscale**			
Item 1: Size	0.67		0.88
Item 2: Private Conversation	0.69		0.91
Item 3: Help	0.68		0.88
**Internal Consistency**			
Cronbach’s α	0.83	0.90	0.95

LSNS-6, the abbreviated version of the Lubben Social Network Scale.

The corrected item-total correlation was to illustrate the correlation between each item and the sum of the other items in the scale. Corrected inter-item correlations using the criterion of 0.3 or higher were used to identify items related to the full scale [22, 23]. As can be seen from Table 3, the items of LSNS-6 were very homogeneous with the coefficients ranging from 0.50 to 0.69. In addition, the coefficients of the item-total subscale correlation ranged from 0.78 to 0.83 for family subscale and from 0.88 to 0.91 for friends subscale. Coefficients of the item-total subscale correlation were necessarily higher than the item-total scale correlation, indicating that stronger homogeneity was observed within the subscales than the LSNS-6.

### Associations between the LSNS-6 and late-life suicidality

Table 4 showed the logistic regression analyses on the associations between late-life suicidality and the LSNS-6, the LSNS-6 family subscale, and the LSNS-6 friends subscale. The results revealed that the increased scores of the LSNS-6 and its family subscale were significantly associated with decreased suicidal risks (ORs<1, *p-*value <0.05). After confounding variables controlled, the significant association between the LSNS-6 family subscale and suicidal outcomes still existed. In contrast, the LSNS-6 friends subscale was not significantly associated with late-life death wishes and suicidal ideation among the mainland Chinese (*p-*value >0.05).

**Table 4:** Logistic regression analyses on the associations between late-life suicidality and the LSNS-6.

Social Network	Model	Death Wishes		Suicidal Ideation	
		*OR*	*95% CI*	*OR*	*95% CI*
LSNS-6	*Crude*	0.94***	(0.91, 0.97)	0.92***	(0.87, 0.97)
	*Adjusted*	1	(0.98, 1.04)	0.96	(0.91, 1.02)
Family subscale	*Crude*	0.89***	(0.84, 0.94)	0.83***	(0.76, 0.90)
	*Adjusted*	0.95*	(0.90, 1.00)	0.87***	(0.80, 0.96)
Friends subscale	*Crude*	0.96	(0.92, 1.01)	0.96	(0.89, 1.03)
	*Adjusted*	1.05	(0.99, 1.10)	1.03	(0.95, 1.12)

OR, odds ratio; CI confidence interval; LSNS-6, abbreviated version of the Lubben Social Network Scale; Adjusted by age, gender, education status, marital status, self-rated financial status, and self-rated health status.

*0.01 ≤ p < 0.05;

** 0.001 ≤ p < 0.01;

***p < 0.001.

## Discussion

From the validation of Chinese version of the LSNS-6, the LSNS-6 showed sufficient internal consistency (Cronbach’s α = 0.832) and two very consistent factor structures (family factor and friend factor), which coincided with the previous findings within the non-Chinese contexts [11, 12]. The present data also demonstrated the LSNS-6 and the two subscales (family subscale and friends subscale) revealed high internal consistency. In sum, the LSNS-6 could be a good tool for assessing social networks among older mainland Chinese.

The present validation study recommends that the LSNS-6 is a good integrated tool to measure social relationships in the Chinese communities. Social relationships are usually classified into categories as structural and functional aspects. Many previous studies conceptualized structural aspects as the existence and interconnections among differing social ties and roles such as social contact, and functional aspects as functions provided or perceived to be available by social connections such as perceived social support and loneliness [6, 7, 24]. The LSNS-6 was constructed from both the structural and functional measurements of social relationships including network size, private conversation, and social support and was significantly correlated with other measurements of social relationships. Therefore, the LSNS-6 is a useful tool to measure social relationships.

The present findings also revealed that assessment of social support networks was essential in working with elderly death wishes and suicidal ideation. As shown in our logistic regression analysis, the LSNS-6 was significantly associated with late-life suicidality. Several studies reported that smaller/ lower / poor / separated social networks were associated with decreased suicide thoughts and behaviors [25–28]. The reasons why social relationships measured by the LSNS-6 are effective factors in late-life suicidality are mainly due to individuals who perceive high levels of available social support are optimistic, and as such, possess a strong sense of self efficacy, positive evaluation of self, low anxiety, and positive expectations on social interactions [29]. In addition, recent research has found that social support appears to be most effective if it is readily accessible and allows the elderly people with suicidal thoughts an opportunity to express themselves [30].

Interestingly, another important finding from the present study revealed that late-life suicidality was highly associated with the LSNS-6 family subscale rather than the friends subscale among the mainland Chinese. This meant that perceived social support from family was more predictive in reducing late-life suicidality than perceived support from friends in the Chinese communities. In western countries however, friends, more than family members, play a defining role in the well-being of older adults [30, 31]. In contrast, family was the most important source of social support followed by friends among the Chinese communities [32]. Social networks include connections with various groups of individuals such as relatives, friends, neighbors and so on. According to the present findings, improving social networks, especially in family bonds, was a promising strategy in reducing late-life suicide risks in mainland China.

For future research or clinical practice, Lubben suggested using a score of less than 12 as a clinical cutoff point of the LSNS-6 to indicate social isolation, which meant, on average, the respondents had less than two people to perform social integration functions. However, further studies would be necessary to validate this proposal for the Chinese communities. The cut point of less than 12 may not be applicable to Chinese older adults. The possible reasons include three aspects. Firstly, social relationships in the Chinese communities play more important roles in late life than in the western communities [4] and Chinese elderly need larger and more interactive social networks to keep social integration, therefore, less than a high score of the LSNS-6 indicates social isolation in the Chinese communities. In addition, it is also possible that when using rating scales, Eastern Asians tend to show more preference for middle values and less preference for extreme small values [33]. Therefore, determining a cutoff score for the Eastern Asian elderly may need to be geared towards a number larger than the 12 points for the western general population. Thirdly, as consistent with the findings that family members are more important to the Chinese than friends, and thus play a defining role in the well-being of older adults, the cutoff score of the LSNS-6 family subscale is higher that of the friends subscale.

This validation however, has several limitations that must be considered in interpreting the findings. At first, a limitation of the present study is that the diagnostic accuracy for social isolation has yet to be developed by this study. Moreover, this study was based on a cross-sectional survey of older adults. One of the major shortcomings of the cross-sectional survey is its inability to capture the changes in the variables over time. Some variables such as age, health and mental health conditions, social network, and others, at the time of the survey might have changed during the 12-month period prior to the survey, which could affect the efficiency of the model [14]. Furthermore, the convergent validity of the LSNS-6 was not successfully examined by our validation study as there were not appropriate comparator instruments included in the database. Some potential comparator instruments of other social relationships in the database were all single-item measurements and without reliability and validity values that have been assessed and reported to date. In addition, the data for this study are drawn from a single administration and so there are no test–retest data. Last but not the least, the random data in this study were extracted from the Beijing subgroup data from the Sample Survey on Aged Population in Urban/Rural China. Thus, caution should be exercised to apply the present findings to the whole aging population in mainland China.

## Conclusion

In sum, our study has several important added values. This present study showed good internal consistency and consistent factor structure of the LSNS-6 as well as its subscales. The present data demonstrated the LSNS-6 could be a good tool for assessing social networks among older mainland Chinese. Most importantly, the present findings also revealed that assessment of social support networks was essential in working with elderly suicidality. Therefore, suggestion is made to improve social networks, especially in family bonds and support, as a promising strategy in reducing late-life suicide risks in mainland China. The increasing size of internal migration within China would, however, reduce family bonds and it could well have much more negative impact on the wellbeing of the elderly population in mainland China.
